# Sexually-dimorphic neurons in the *Drosophila* whole-brain connectome

**DOI:** 10.21203/rs.3.rs-6881911/v1

**Published:** 2025-06-26

**Authors:** David Deutsch, Arie Matsliah, Kaiyu Wang, Sven Dorkenwald, Arpita Mondal, Austin Burke, James Hebditch, Jay Gager, Szi-Chieh Yu, Amy Sterling, Claire McKellar, Philipp Schlegel, Stephan Gerhard, Gabriella Sterne, Marta Costa, Katharina Eichler, Yijie Yin, S.X.E. Gregory, Barry Dickson, H. Sebastian Seung, Mala Murthy

**Affiliations:** 1 Department of Neurobiology, University of Haifa, Haifa, Israel; 2 Princeton Neuroscience Institute, Princeton University, Princeton, NJ, USA; 3 Shanghai Center for Brain Science and Brain-Inspired Intelligence Technology, Shanghai, China; 4 Allen Institute for Brain Science, Seattle, WA, USA; 5 Neurobiology Division, MRC Laboratory of Molecular Biology, Cambridge, UK; 6 Aware LLC, Rappenstrasse, Effretikon, Switzerland; 7 Drosophila Connectomics Group, Department of Zoology, University of Cambridge, Cambridge, UK; 8 University of Rochester Medical Center, Department of Biomedical Genetics, New York, NY USA; 9 Queensland Brain Institute, University of Queensland, Brisbane, Australia; 10 Computer Science Department, Princeton University, Princeton, NJ, USA

## Abstract

Sexual dimorphisms are present across brains. Male and female brains contain sets of cell types with differences in cell number, morphology, or synaptic connectivity between the two sexes. These differences are driven by differentially-expressed transcription factors, which set the stage for disparate sexual and social behaviors observed between males and females, such as courtship, aggression, receptivity, and mating. In the *Drosophila* brain, sexual dimorphisms result from differential expression of two transcription factors, Fruitless (Fru) and Doublesex (Dsx), and genetic reagents driven by enhancers for Fru and Dsx label sexually-dimorphic neurons in both male and female brains. The recent release of the first whole-brain connectome for *Drosophila* provides a unique opportunity to study the connectivity between these neurons as well as their integration into the larger brain network. Here, we identify 91 putative Fru or Dsx cell types, comprising ~1400 neurons, within the whole-brain connectome, using morphological similarity between electron microscopic (EM) reconstructions and light microscopic (LM) images of known Fru and Dsx neurons. We discover that while Fru and Dsx neurons are highly interconnected, each cell type typically receives more inputs from and sends more outputs to non-Fru/Dsx neurons. We characterize the connectivity in the Fru/Dsx networks to predict the function of cell types not previously characterized, we measure distances to the sensory periphery and uncover multisensory interactions, and we map connections to descending neurons that drive behavior. All Fru and Dsx labels reported here are shared within FlyWire Codex (codex.flywire.ai; gene==Fruitless or Doublesex); this work is a critical first step towards deciphering the neural basis of sexually-dimorphic behaviors and for making comparisons with future connectomes of the male brain.

## Introduction

In *Drosophila melanogaster*, the transcription factor Fruitless is transcribed in both males and females, but Fruitless protein is only produced in males, while males and females express sex-specific isoforms of the transcription factor Doublesex (Rideout et al., 2007; Dickson, 2008; Kimura et al., 2008). Neurons that transcribe Fruitless and Doublesex can be labeled in both males and females (Kimura et al., 2005; Cachero et al., 2010; Yu et al., 2010; Liu, 2012; Zhou et al., 2014; Wohl et al., 2020; Brovkina et al., 2021; Palmateer et al., 2023), revealing the population of neurons underlying sex-specific behaviors, such as courtship (Rideout et al., 2007; von Philipsborn et al., 2011; Clowney et al., 2015; Rezával et al., 2016; Deutsch et al., 2019; Roemschied et al., 2023), receptivity (Zhou et al., 2014; Wang et al., 2021), copulation (Pavlou et al., 2016; Kerwin et al., 2020; Mezzera et al., 2020), aggression (Vrontou et al., 2006; Certel et al., 2007; Deutsch et al., 2020; Schretter et al., 2020; Wohl et al., 2020; Chiu et al., 2023), and egg laying (Rezával et al., 2012, 2014; Wang et al., 2020b; Vijayan et al., 2022, 2023). While targeted genetic reagents have enabled studying specific cell types within this population, and connecting those cell types to behavior, we still know relatively little about the connectivity of Fruitless and Doublesex cell types across the brain of either males or females. Prior work indicated that Fruitless-expressing neurons preferentially connect to each other in order to process sex-specific information and effectively drive sex-specific behaviors (Cachero et al., 2010; Yu et al., 2010). Indeed, most studies of specific Fruitless or Doublesex cell types focus only on known connectivity with other Fruitless and Doublesex cell types. Here, we test this hypothesis by identifying a large population of putative Fruitless and Doublesex neurons in a female whole-brain connectome (Dorkenwald et al., 2023; Schlegel et al., 2024), and characterizing connectivity at brain-scale. Identifying these neurons relies on comparisons between light microscopic (LM) images of Fru and Dsx neurons (Cachero et al., 2010; Yu et al., 2010; Chiang et al., 2011; Liu, 2012; Wu et al., 2016; McKellar et al., 2019; Wang et al., 2020b, 2021; Nojima et al., 2021; Schlegel et al., 2024; Stürner et al., 2024) with electron microscopic (EM) reconstructions; this is possible because *Drosophila* neurons have stereotyped morphologies (Schlegel et al., 2024). We semi-manually conducted this matching, using tools for registering LM and EM datasets (see Methods; (Costa et al., 2016; Matsliah et al., 2023)), identifying a population of 1278 Fru neurons, 50 Dsx neurons and 79 neurons with morphology that fits both a Fru type and a Dsx type, that we labeled as Fru-Dsx (Fru pMP3, pMP5 have shared morphology with Dsx pCd1, pCd2 (Kimura et al., 2015), respectively). We map connectivity patterns, showing that Fru/Dsx neurons are more interconnected than spatially matched controls, but still mostly connected to non-Fru/Dsx partners. We discover a “core network” of strongly interconnected Fru/Dsx neurons and use connectivity to predict the function of new cell types. Finally, we highlight multisensory integration and connections to descending motor command neurons. This study extends the connectomic landscape of sexual dimorphism beyond previous Fru/Dsx circuit work, and provides a resource for future functional investigations of female sexual and social behaviors.

## Results

### Identifying Putative Fruitless or Doublesex neurons in the Female Whole-Brain Connectome

**Figure 1.**
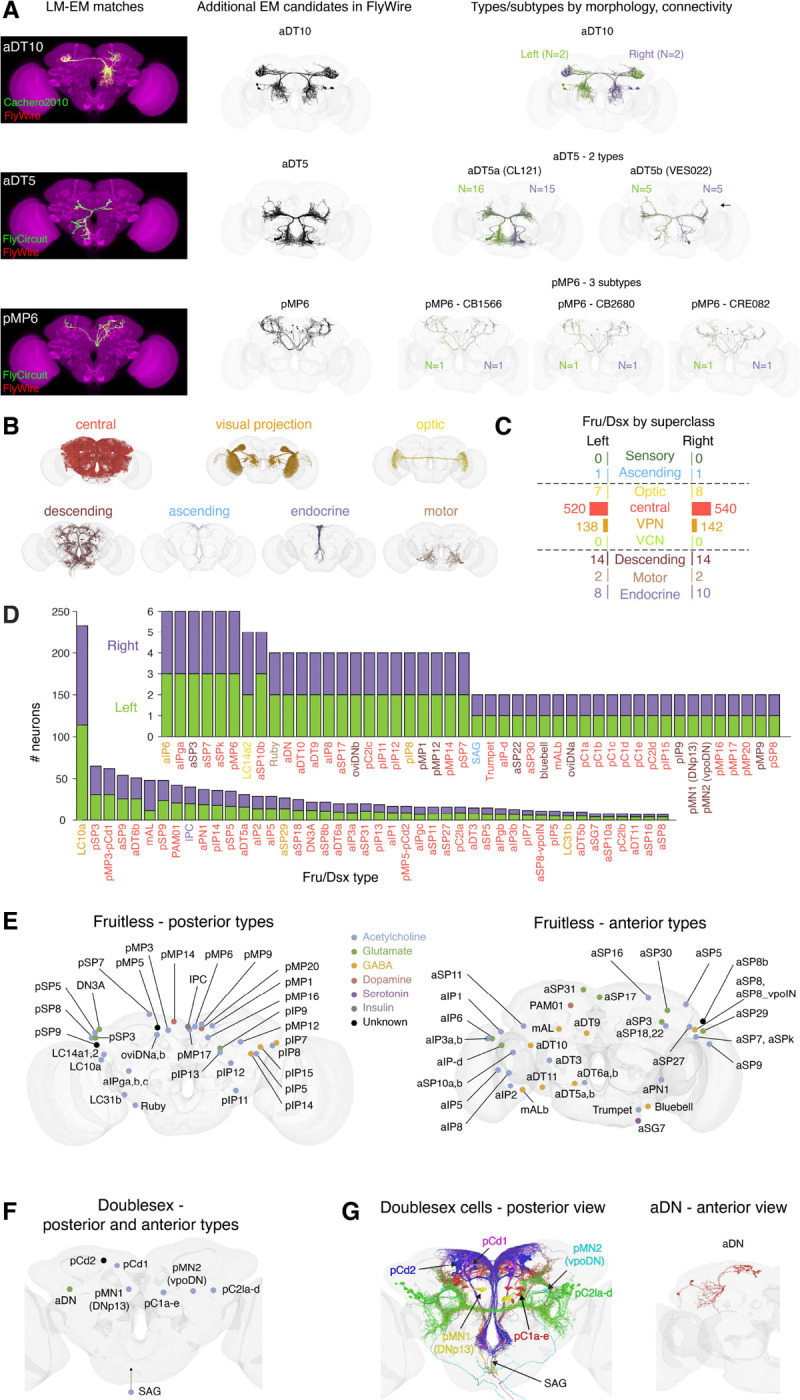
Identifying putative Fruitless and Doublesex (Fru/Dsx) neurons in the FlyWire brain connectome **(A)** FlyWire (EM) candidates of Fru/Dsx types were found by direct comparison to light microscopy (LM) stacks of Fru/Dsx neurons (single neuron ‘clones’ or sparse neuron labeling with genetic lines (Cachero et al., 2010; Yu et al., 2010; Chiang et al., 2011; Liu, 2012; Wu et al., 2016; McKellar et al., 2019; Wang et al., 2020b, 2021; Nojima et al., 2021; Sterne et al., 2021; Court et al., 2023)) or via matching to previously identified Fru/Dsx candidates in the hemibrain dataset (Scheffer et al., 2020; Schlegel et al., 2024). In both cases, FlyWire candidates were compared with all available sources (see Table 1). Additional Fru/Dsx candidate neurons in FlyWire were identified based on existing annotations of FlyWire neurons in Codex (Matsliah et al., 2024; Schlegel et al., 2024); see Methods). For a given LM source there may by a single Fru/Dsx type (e.g., aDT10; top row), or multiple types that both overlap with the LM morphology, but are not similar in morphology (e.g., Fru/Dsx aDT5a and aDT5b; middle row). Since Fru/Dsx types are defined only by morphology, a given Fru/Dsx may comprise multiple FlyWire subtypes that differ by their connectivity (e.g., pMP6; bottom row). There are a total of 91 Fru/Dsx types and 236 subtypes in the dataset. **(B)** We identified 1407 putative Fru/Dsx cells in total, here sorted by ‘superclass’ (Dorkenwald et al., 2024) and rendered to a brain template. Our dataset excludes y Fru/Dsx neurons in the mushroom body (see Methods), and in the following superclasses: sensory and visual centrifugal (see codex.flywire.ai for more information on superclasses). **(C)** Number of Fru/Dsx cells in each superclass and hemisphere. **(D)** Number of cells per type in the left (green) and right (purple) hemispheres. Type names are colored by superclass (as in B-C). **(E)** Soma clusters of putative Fruitless types in the posterior (left) and anterior (right) brain, colored by known or predicted (Eckstein et al., 2024) neurotransmitter expression. When classifier prediction did not agree on at least 60% of individual neurons, the type was colored back. **(F)** same as in (E), but for all Doublesex types, in posterior view (all Dsx cells in the brain are posterior, except aDN). The Dsx ascending SAG neurons are marked outside the brain (not at their physical location) with an upward arrow. Fru pMP3 and Dsx pCd1 share morphology, as do Fru pMP5 and Dsx pCd2. Because we are unable to distinguish these types in the connectome, they are referred to as pMP3/pCd1 and pMP5/pCd2. **(G)** All posterior (left) and anterior (right) putative Dsx neurons rendered to a brain template and colored by type. aDN neurons (2 per hemisphere) are shown only in the left hemisphere.

**Figure 2.**
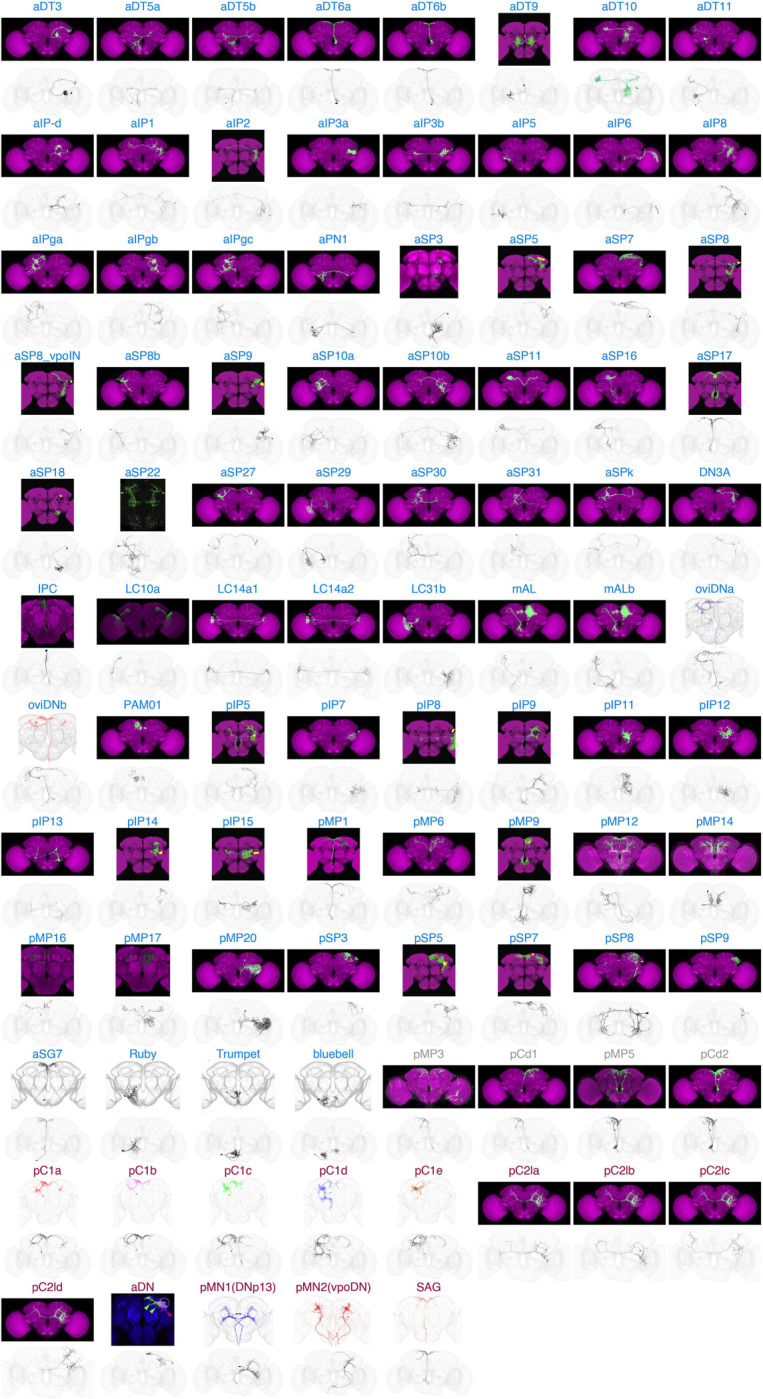
LM-to-EM matches for each Fru/Dsx type For each one of the 91 Fru/Dsx types, a light microscopy (LM) image (top) and electron microscopy (EM) reconstruction from FlyWire (bottom) is shown - for EM images, we chose a single example cell. The LM source for each type and a FlyWire link corresponding to the displayed neuron are provided in Table 1. In two cases a single LM image was used for multiple Fru/Dsx types: pC2l (pC2la, pC2lb, pC2lc, pC2ld) and LC14a (LC14a1, LC14a2). Several Fru/Dsx types have additional subtypes (based on annotations in Codex) - see Table 1 and Fig. 3. Fru/Dsx types are ordered as in Table 1 (first Fru, then Fru/Dsx, and then Dsx). Cell type name is colored as elsewhere: Fru in blue, Dsx in red, and Fru-Dsx (Fru/Dsx types pMP3-pCd1 and pMP5-pCd2; for those types there is shared morphology between a Fru and a Dsx type) in gray. For cell type aSP30, the source image is not from LM, but rather a hemibrain segment that was previously identified as aSP30 (ID 800579317). This image is marked with *.

To identify Fruitless (Fru) or Doublesex (Dsx) neurons (hereafter referred to as Fru/Dsx) in a whole-brain connectome of *Drosophila* (Zheng et al., 2018; Dorkenwald et al., 2024; Schlegel et al., 2024), we compared light microscopy (LM) images of single Fru or Dsx neurons or sparse genetic driver lines previously shown to label Fru or Dsx neurons (Cachero et al., 2010; Yu et al., 2010; Chiang et al., 2011; Liu, 2012; Kimura et al., 2015; Wu et al., 2016; McKellar et al., 2019; Wang et al., 2020b, 2021; Nojima et al., 2021; Sterne et al., 2021; Stürner et al., 2024) to electron microscopy (EM) reconstructions of single neurons in the connectome (Fig. 1A; see Methods and Table 1) either manually or using candidates from automated comparison of color-depth maximum intensity projections (MIP) (Cachero et al., 2010; Chiang et al., 2011; Otsuna et al., 2018), downloaded from www.virtualflybrain.org (Court et al., 2023) (see Methods). When available, we used previously labeled Fru/Dsx candidates in the ‘hemibrain’ dataset (Scheffer et al., 2020) as seeds for finding candidates in the whole-brain connectome by using FlyWire gateway for transformation (https://flywiregateway.pniapps.org). Often the hemibrain segments were missing a fraction of the cell (Supp. Fig. 1S1), making it challenging in that dataset to correctly subtype neurons. Additional Fru/Dsx candidates were then found in FlyWire using similarity in morphology or connectivity, as well as available annotations in Codex (Matsliah et al., 2023; Dorkenwald et al., 2024; Schlegel et al., 2024). In all cases, the final candidates were compared to LM images (see Table 1). Combining all available sources, we identified a total of 1407 putative Fru/Dsx cells in the whole-brain connectome, from 7 super classes and in both hemispheres (Figs. 1B–C and Fig. 2). These neurons make up 91 Fru/Dsx types, with roughly the same number of cells per hemisphere for each type (Fig. 1D). In many cases, a given LM cell type (by morphology) corresponds to multiple possible cell types in the connectome (a connectome cell type is largely defined by connectivity (Matsliah et al., 2024; Schlegel et al., 2024)), resulting in multiple ‘subtypes’ for a single Fru/Dsx type and a total of 236 subtypes. We include all EM candidates (Supp. Fig. 1S2), preferring false positives over false negatives. We indicate these connectome-based subtypes (see Table 1) in our analysis (e.g., the descending Fru cell type pMP12 comprises two connectome-based cell types: DNp60 and DNp67, resulting in subtypes pMP12-DNp60 and pMP12-DNp67). Our dataset consists of 1278 Fru neurons of 195 connectome-based cell types and 50 Dsx neurons of 16 subtypes (Fig. 2). In two cases, Fru and Dsx types have shared morphology: Dsx pCd1 neurons share morphology with Fru pMP3, and Dsx pCd2 neurons share morphology with Fru pMP5 (Supp. Fig. 1S2). These two populations are relatively heterogeneous in terms of connectivity, with 15 and 8 connectomic cell types for pCd1/pMP3 and pCd2/pMP5, respectively. As our detection of Fru and Dsx cells is based solely on morphology, we group these types together in our analyses. All of our labels have been available in Codex (codex.flywire.ai) since the release of the connectome dataset last year (Dorkenwald et al., 2024).

**Figure 3 -:**
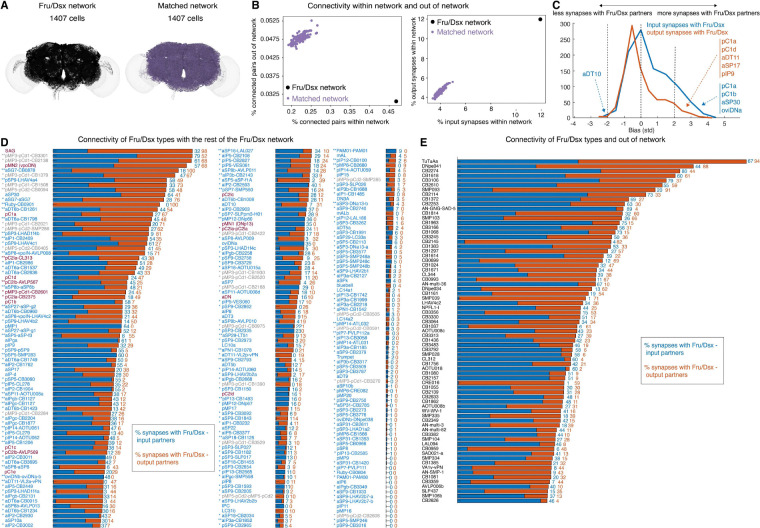
Connectivity in the Fru/Dsx network **(A)** All 1407 putative Fru/Dsx neurons (black), and a set of 1407 neurons from one example ‘matched network’ (purple). ‘Matched networks’ (see Methods) have the same superclass and spatial distribution as the Fru/Dsx neurons. **(B)** (left) Fru/Dsx neurons (black) make more connections within-network (versus out-of-network), as compared with 100 matched networks. A 5 synapse minimum threshold for a connection was applied. (right) Fru/Dsx neurons (black) have more input and output synapses within-network, as compared with 100 matched networks (purple). Synapses within a primary type were excluded for both Fru/Dsx and matched networks, to avoid possible bias for within network connectivity due to more cells per type in the Fru/Dsx network compared to a matched network. **(C)** Synaptic Input (blue) and output (red) bias distributions, measuring the bias of each Fru/Dsx type to form more (positive bias) or less (negative bias) synapses with Fru/Dsx partners compared to the number of synapses with any of its partners (see Methods). The input and output biases were calculated separately for each individual Fru/Dsx cell, and then averaged for all the cells of a given type. For example, pC1a has a strong bias for connections with Fru/Dsx partners for both inputs and outputs, while for pC1d, the strong bias is only for the output connections. **(D)** The percentage of synapses each Fru/Dsx subtype has with any Fru/Dsx partner (including within type), for inputs (synapses with presynaptic partners; blue) and outputs (postsynaptic partners; red). When a cell type has multiple subtypes, * or ** appear near the subtype name. ** indicates that for a given type, this subtype has the most connections with other Fru/Dsx neurons. For example, pMP3/pCd1-CB3301 has 79% of input synapses from other Fru/Dsx neurons and 52% output synapses, more than the input+output % for any other subtype of pMP3/pCd1. When a given Fru/Dsx type has multiple subtypes but some cells do not have a defined primary type in Codex, the Fru/Dsx type is used also as the subtype name (e.g., ‘pSP9-pSP9’). This is in contrast to cases where there is only one primary type per Fru/Dsx type (e.g., pC1a or aSP30) that are not marked with an asterisk. The total number of synapses per type and the number of Fru/Dsx synapses per type both exclude within-subtype connections. **(E)** Same as (D), but for FlyWire cell types not in the Fru/Dsx list, that have a large proportion of connections with Fru/Dsx neurons (50% or more). These cell types are candidates for being Fruitless or Doublesex positive.

The total number of Fru/Dsx neurons in the female brain is not known. Prior work estimates 1500–2000 neurons (Lee et al., 2000; Cachero et al., 2010; Yu et al., 2010) or roughly 2% (Stockinger et al., 2005) of the neurons in the female brain are Fru and a few tens are Dsx (Kimura et al., 2015; Rezával et al., 2016); based on this, we have likely identified just over 50% of the Fru/Dsx neurons in the female connectome. This underrepresentation is due to i) exclusion of both sensory neurons and mushroom body Kenyon cells, as we could not unambiguously identify the Fru subsets among them (similar to what was done in (Yu et al., 2010)), ii) exclusion of many optic lobe neurons that were similarly difficult to distinguish Fru from non-Fru (e.g., the medulla ‘M neuron’ (Chiang et al., 2011; Tanaka et al., 2017)), and iii) exclusion of putative Fru/Dsx types for which we did not have LM images (including cells with sexual dimorphism in the female and male optic lobes (Matsliah et al., 2024; Nern et al., 2025)). We also include only two ascending neurons (the Dsx-positive SAG neurons (Figs. 1B–C)), missing known ascending Fru neurons such as dMS6 (Yu et al., 2010; Liu, 2012). Cell type “AN-multi-82” (with 44% and 11% of its input and output synapses with Fru/Dsx; see Fig. 3D) contains two cells, one of them strongly overlapping with ascending dMS6 (Liu, 2012), while the other is missing a contralateral branch in FlyWire and therefore the pair are excluded from our list. Because the FlyWire brain connectome ends in the neck connective, a full CNS connectome will be necessary to identify more Fru/Dsx ANs with cell bodies and dendrites in the nerve cord (Stürner et al., 2025). Finally, once a male brain connectome is available, comparison of the male and female brain connectomes can identify additional sex-specific neurons or neurons with sex-different connectivity. However, with only a single full brain connectome of each sex, it will be challenging to distinguish across-individual variation from sex-specific variation. In that case, comparison with LM images of Fruitless and Doublesex neurons will be valuable. We therefore view our approach as complementary to the approach that compares two connectomes. Further experimental work will be needed to refine and extend the cell types presented here.

### Positions of Fruitless and Doublesex Neurons

Many Fru/Dsx cell types were named previously (Kimura et al., 2008; Cachero et al., 2010; Rideout et al., 2010; Yu et al., 2010; Liu, 2012; Wang et al., 2021), and we used the existing names when possible (see Table 1), only adding new names based on prior ones. For example, aSP30 is a previously uncharacterized group (see Fig. 6 for proposed function), but its cell bodies are in the vicinity of other aSP Fru types, such as aSP3 (Yu et al., 2010) and aSP18 (Liu, 2012). There are 36 Fru cell types with somas in the posterior brain ((Fig. 1E); this includes pMP3,5 that overlap in morphology with pCd1,2), and 42 Fru cell types with somas in the anterior brain (Fig. 1F). In addition, there are 15 Dsx cell types, including the ascending SAG neurons (Figs. 1F–G). All Dsx somas are close to the posterior surface, except the more anterior aDN somas (Fig. 1G). While we couldn’t distinguish between Dsx pCd1 and Fru pMP3 or between Dsx pCd2 and Fru pMP5 (Supp. Fig. 1S2), they are presented separately in the Fru and Dsx soma-location maps (Fig. 1E–G). The Dsx types pMN1/pMN2 (Kimura et al., 2015; Deutsch et al., 2019) have identical morphology to the DNp13/vpoDN types (Wang et al., 2020a, 2021), respectively. The division of pC1 cells into subtypes was previously reported (Deutsch et al., 2020; Wang et al., 2021), while the division of pC2l is new here (see Fig. 6).

### Fruitless and Doublesex Connectivity in the Whole-Brain Connectome

We created an LM-EM comparison catalog for each Fru/Dsx type (Fig. 2; note that often there is more than a single LM source, see Table 2), along with an ‘ID card’ for each type (Supp. Fig. 2S1). Each card contains information about cell morphology, known or predicted neurotransmitter expression (Eckstein et al., 2024), and top inputs and outputs in the whole-brain connectome (from any cell type). This information also exists in Codex (https://codex.flywire.ai/), where identified Fru/Dsx cells are annotated as having ‘Gene Expression’ equal to ‘Fruitless’ or ‘Doublesex’ (or both in the case of pMP3/pCd1 and pMP5/pCd2).

### Connectivity Within the Fru/Dsx Network

Prior work on individual Fru or Dsx cell types has focused mostly on connections with other Fru/Dsx neurons (von Philipsborn et al., 2011; Deutsch et al., 2020; Jung et al., 2020; Schretter et al., 2020; Wang et al., 2021; Laturney et al., 2023). This focus has led to an assumption that Fru/Dsx neurons preferentially connect to each other to detect sex-specific signals and drive sex-specific behaviors (Yu et al., 2010). We test this assumption first by building ‘matched networks’ - random sets of neurons in the whole-brain connectome that match the spatial and super class distributions of Fru/Dsx neurons and with the same total number of cells (Fig. 3A and Fig. 3S1; see Methods). First, we measured the fraction of synapses between each cell (separately for input synapses and output synapses) and other cells in its network, out of all synapses with any partner in the connectome (Fig. 3B, left). Second, we compared the fraction of connected cell pairs in each network out of all possible pairs (Fig. 3B, right). Both measures indicate that Fru/Dsx cells are significantly more interconnected than expected relative to their physical proximity as measured by distance between their cell bodies.

We next asked whether Fru/Dsx neurons have stronger connections with their Fru/Dsx partners (out of all of their synaptic partners). For each individual cell, we compared the number of synapses (separately for inputs and outputs) with a Fru/Dsx partner, and the number of synapses with the same number of partners chosen at random from all partners (Fig. 3C; see Methods). We uncovered a positive bias for both inputs and outputs of Fru/Dsx neurons - that is, most Fru/Dsx neurons make stronger connections with other Fru/Dsx neurons.

There is heterogeneity, however; some cell types have a very strong positive bias for both their inputs and outputs (e.g., pC1a), some have a strong positive bias for only their inputs or outputs (e.g., pC1b and pC1d), and a single type (aDT10) has a negative bias. See Fig. 2S1 (ID cards) for more information about the top input and output cell types for each Fru/Dsx neuron. This heterogeneity can be better appreciated by plotting the fraction of input and output synapses from the Fru/Dsx network for every Fru/Dsx neuron we identified (Fig. 3D). When there are multiple subtypes for a given Fru/Dsx type, we distinguish between them - the subtype with the most connections to other Fru/Dsx neurons is marked with a double asterisk (**), and the remaining subtypes with a single asterisk (*). Based on the observation that Fru/Dsx neurons are strongly interconnected (Fig. 3A–C), this analysis may indicate which subtypes of a given Fru/Dsx type are more likely to be Fruitless or Doublesex expressing. As one example, pMP3-pCd1 (meaning, pMP3 or pCd1) and pMP5-pCd2 (pMP5 or pCd2) have 62 and 17 cells, respectively. The number of Dsx pCd1 and pCd2 are estimated as 8 and 4 (Kimura et al., 2015; Imoto et al., 2024) (note that Dsx pCd neurons are also termed ‘pC3’ (Rideout et al., 2010; Rezával et al., 2016)). pCd2 neurons are involved to link post-mating sugar with increased appetite by connecting pC1a neurons (which carry information about mating state) to Fru Lgr3-expressing cells (Laturney et al., 2023). Lgr3-expressing cells (Meissner et al., 2016; Laturney et al., 2023) have similar morphology as aDT6a,b, and are likely a subset of aDT6. Only 4 pMP5-pCd2 cells are directly downstream pC1a and directly upstream aDT6a,b: subtypes SMP286 and CB0405, making them good candidates for being Dsx pCd2 cells. Interestingly, the pMP5-pCd2 subtypes that are most connected to Fru/Dsx are also CB0094 and SMP286 (Fig. 3D).

The most connected Fru/Dsx types (as defined by percent of input and output synapses; Fig. 3D) are the Dsx ascending SAG neurons (98% output synapses with other Fru/Dsx neurons), the descending Dsx pMN2 (vpoDN) cells (57% input and 68% output synapses from other Fru/Dsx), and two of the pCd1/pMP3 subtypes. At the other extreme, Fru pIP11 neurons have only 1% of their input and output synapses with other Fru/Dsx neurons, while Fru pMP16 has 0%. For 15 of 91 cell types we have lower confidence in the LM-EM match (Table 1). This includes cases where the cell type was small and without a clear distinguishable structure (e.g., pIP7) or when some part of the cell was missing in the LM or EM side (e.g., pIP14). Examining connectivity with the rest of the Fru/Dsx network suggests that of those 15 types, 6 have strong connectivity to other Fru/Dsx types (aIP5,aIP-d, aIP3b, pIP14,aSP5, pSP5), 2 have intermediate connectivity (PAM01 and pSP3) and 7 have weak connectivity (pIP7,11,12, aIP3a, DN3A, aSP10b and IPC). The average connectivity between Fru/Dsx neurons is 17% for inputs and 15.5% for outputs. In fact, most neurons in our list have less than half of their synaptic connections with other Fru/Dsx neurons. While this may be due in part to our missing some Fru/Dsx neurons, it highlights an important point - all Fru/Dsx neurons make connections (and in most cases the majority of their connections) outside of the putative Fru/Dsx network. There are few studies on the connections between Fru/Dsx neurons and non-sexually-dimorphic neurons, and the function of these connections largely remains to be determined. For example, a pair of neurons called ‘LC062’ drives female aggression (Tao et al., 2024) and are not identified as Fru/Dsx, but here we find that these cells form direct connections with a number of Fru/Dsx types including Dsx pC1d, Fru aIPgc and Fru pMP12 (descending types DNp60, DNp67). Finally, we identified neurons in the connectome that are not labeled as Fru/Dsx, but that make a large percentage of their synapses with Fru/Dsx neurons (Fig. 3E). This was done in an effort to identify putative Fru/Dsx we may have missed, and to also identify non-Fru/Dsx neurons with important relevance to sexual and social behaviors in *Drosophila*. The most-connected type in this list is TuTuAa (with 94% output synapses with Fru/Dsx; most of these connections are with the numerous Fru LC10a neurons). This cell type was recently shown to be involved in enabling visually-guided aggressive encounters (Schretter et al., 2025). However, the other types at the top of the list have not yet been functionally characterized (e.g., DNpe041 is a DN identified in the EM dataset and not identified before by LM (Stürner et al., 2025)) and other top neurons are annotated as ‘CB’ for central brain).

### Connectivity-Based Clustering of Fru/Dsx Neurons

**Figure 4 -:**
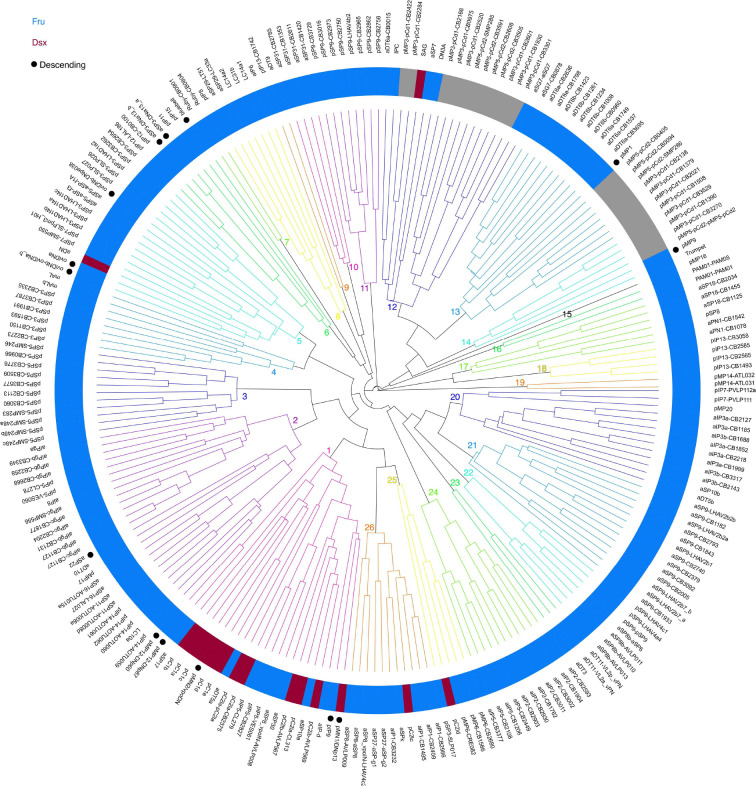
Connectivity-based clustering of Fru/Dsx neurons Fru/Dsx neurons were clustered into 26 clusters based on their connectivity (input and output) with all cell types in the whole brain conenctome (see Methods). Cluster colors are arbitrary, except that neighbouring clusters are assigned different colors. Fru cell types are indicated in blue, Dsx cell types in dark red, and Fru/Dsx cell types in gray. Consistent with previous clustering into subtypes (Matsliah et al., 2023; Schlegel et al., 2024), cells from the same primary type (or Fru/Dsx subtype) share the same cluster. A black filled circle indicates a descending type.

We next clustered the Fru/Dsx neurons by connectivity to all neurons in the connectome (Fig. 4). A similar approach was used previously to identify functional groupings among optic lobe neurons in the connectome (Matsliah et al., 2024). Clustering was done based on pairwise distances between connectivity feature vectors, representing the total number of input and output synapses this type has with all the FlyWire cell types. With a cut at distance 0.3 (see Methods) we find 26 clusters. Clustering groups similar subtypes, as expected. For example, mAL and mALb group together in cluster 4. mALb is a new Fru type - it shares morphology with mAL and this analysis reveals that the connectivity of both types is also similar.

We find that Dsx cell types largely cluster together (cluster 1) - these cell types, along with several of the Fru cell types in the same cluster, are explored further in Figure 6. Previously we studied the auditory responses of Dsx pC2l neurons, a subset of which show strong tuning for courtship pulse song (Deutsch et al., 2019). This tuning is similar to the responses of DNp13 and asp10 (Baker et al., 2022). We had observed that only a subset of pC2l neurons responded to song (Deutsch et al., 2019). Based on the clustering results here, we posit that pC2la and pC2lb, which cluster with DNp13 and asp10 in cluster 1, are the likely song-responsive subsets. Fru pIP5 neurons share morphology with pC2l (Fig. 2), and also are grouped into cluster 1, suggesting they may also share pulse song responses (not yet tested). pC2lc and pC2ld are in separate clusters (clusters 26 and 25, respectively) and therefore may not be song responsive (although pC2lc clusters with aIP1-CB2409 and CB1485, that have low rank distance from both gustatory and mechanosensory neurons - see Figure 7).

aIPg neurons that drive female aggression (Deutsch et al., 2020; Schretter et al., 2020) cluster together with LC10a neurons (previously shown to be recruited for visual-based chasing in females during aggression (Schretter et al., 2025)), as well as several other Fru cell types all in cluster 2, which we posit are also involved in aggression, including aSP22 (a sexually dimorphic DN that drives male sexual behaviors but which has not yet been studied in females (Stürner et al., 2025)) and aDT10, which links the LC10a neurons to circuits in the LAL that drive locomotion important for chasing (Hindmarsh Sten et al., 2021). The descending types DNp60 and DNp67 are also included in cluster 2 - we explore their putative function more in Figure 7.

Fru pIP9 and Dsx pMN1/DNp13 share overlapping connectivity, consistent with a possible role of pIP9 in controlling female rejecting behavior (see also Figure 6). More broadly, we find that DNs are distributed throughout different clusters, suggesting that each cluster has its own conduit to drive the premotor circuits in the VNC.

We find that clusters also sort by sensory modality (e.g., clusters 20–22 contain neurons of the olfactory pathway), and this is explored more in Figure 7. Fru visual projection neurons, other than LC10a, group together in clusters 8 and 9. Proximate to these clusters are taste neurons (cluster 7) and aSP31 neurons (cluster 10), a new Fru cell type whose function is unknown. aDN and oviDN group together in cluster 5, consistent with their role in controlling female oviposition (Nojima et al., 2021). Lastly, pMP5-pCd2 (inhibitory) is clustered with aDT6a,b, consistent with their shared circuitry (see (Laturney et al., 2023) and discussion above) and with pMP3-pCd1 (excitatory). Both aDT6 and pCd1 were shown to respond to pheromones and are involved in controlling social behaviors (Fan et al., 2013; Zhou et al., 2014; Jung et al., 2020).

### Network Layout of Fru/Dsx Neurons

**Figure 5 -:**
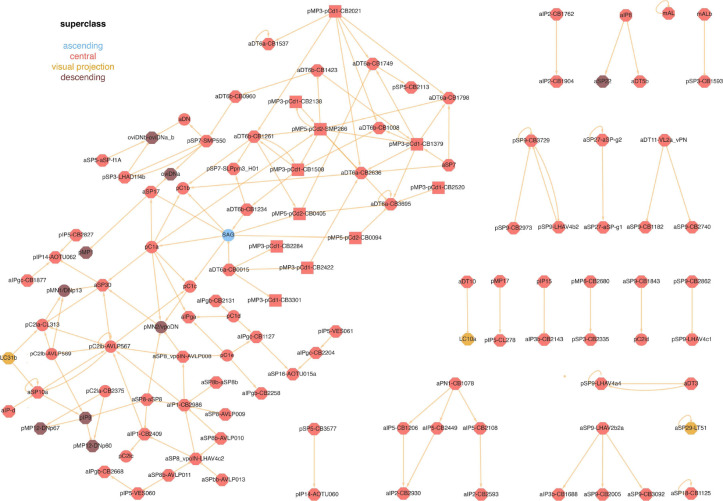
Network of significant connections between Fru/Dsx subtypes Network diagram for Fru/Dsx types. Edge from type A to type B is shown only if at least 3% of A’s output synapses are to B cells *and* at least 3% of B’s input synapses are from A cells (‘symmetric threshold’, see Methods). Edge widths further represent the absolute count of synapses connecting the types, and edge tips represent excitation/inhibition (arrow=excitation, round=inhibition) based on neurotransmitter prediction (Cholinergic cells are considered excitatory, while GABAergic and Glutamatergic cells are considered inhibitory). Node colors represent superclasses, and their shapes represent genes (ellipse=Dsx, octagon=Fru, rectangle=Dsx or Fru, hexagon=other). 119 out of 236 Fru/Dsx subtypes are present in the network.

**Figure 6 -:**
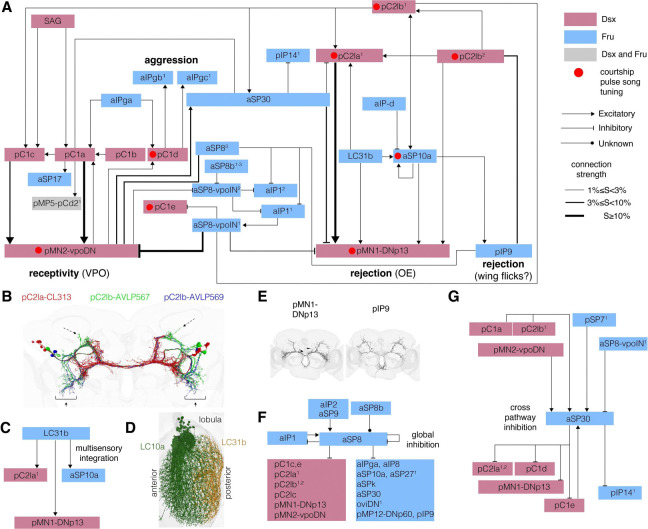
Predicting Function from Connectivity **(A)** Strongest direct and indirect connections of pMN1-DNp13 and PMN2-vpoDN types with Fru/Dsx types. First, the subgroup of Fru/Dsx types with a strong (1% symmetric threshold; see Methods) directly connected to pMN1,2 were added. Then, the Fru/Dsx types connected to this subgroup (using a 3% symmetric threshold) were added. Line width reflects connection strength (S), using three categories for the strength. Types that are known to be tuned to ‘Pulse song’ (one of the two major courtship song types in *D. melanogaster*) are marked with a red box. None of the types in this diagram is known to be tuned to ‘Sine song’. Excitatory and inhibitory connections are represented by pointed and flat arrows, respectively. Connections are considered inhibitory if predicted in FlyWire as GABAergic or glutamatergic, and as excitatory if predicted as cholinergic, with the following caveat: SAG neurons are predicted to be Serotonergic in FlyWire but are shown to be cholinergic (Wang et al., 2020b). ‘Fru’, ‘Dsx’ and ‘Fru or Dsx’ are colored in blue, red and gray as done elsewhere. **(B)** Three pC2lb subtypes that appear in (A) are rendered to brain template in FlyWire: pC2la-CL313 (red), pC2lb-AVLP567 (green) and pC2lb-AVLP569 (blue). Arrows point to processes that exist in pC2lb but not in pC2la. Dashed arrows - small posterior-lateral processes that exist in pC2lb-AVLP567 but not in pC2lb-AVLP569. Note that for simplicity, only direct connections with LC31b are shown, and not connections between the types that are connected to LC31b, even if they are strongly connected with each other. **(C)** Direct connections between Fru LC31b and all Fru/Dsx types. A symmetric threshold of 0.05% was used, lower than the threshold used in (A). LC31b is a visual projecting neuron that is directly connected upstream with Fru+ and Dsx+ auditory neurons that are tuned to Pulse-song. This suggests multisensory integration in the rejection-controlling circuit. Note that for simplicity, only direct connections with LC31b are shown, and not connections between the types that are connected to LC31b, even if they are strongly connected with each other. **(D)** Innervation of the anterior and posterior lobula by example LC10a and LC31b cells. **(E)** The descending types Dsx pMN1-DNp13 and Fru pIP9 are rendered in FlyWire and colored in green (Dsx+) or purple (Fru+). Note that as those are descending neurons, the ventral part, in the VNC, is missing in our images. Each type has exactly 1 cell per hemisphere. pMN1-DNp13 has shared morphology (as well as shared connectivity, see Fig 4, Fig 5 and Fig. 6A) with pIP9, possibly also sharing functional roles. **(F)-(G)** Direct connections between aSP8 or aSP30 in panel F and G, respectively and all Fru/Dsx types. A symmetric threshold of 0.05% was used as in (C). **(F)** aSP8 inhibits both pMN1-DNp13 and pMN2-vpoDN and cells that are connected directly to those two types, therefore possibly driving ‘global inhibition’ in the rejection and receptivity. **(G)** aSP30 receives excitatory inputs from pMN2-vpoDN (which controls a receptivity female behavior, vaginal plate opening) and from other cells types which are strongly connected to pMN2-vpoDN, and sends inhibitory connections to pMN1-DNp13 (which controls a rejecting female behavior, ovipositor extrusion) and to cells that are strongly connected to pMN1-DNp13, therefore driving ‘cross inhibition’. aSP8-vpoIN^1^ = AVLP008; aSP8-vpoIN^2^ = LHAV4c2; aSP8^3^ = aSP8; aSP8b^1–3^ = AVLP010,011,013; aIPgb^1^ = CB2131; aIPgc^1^ = CB1127; aIP1^1^ = CB2986; aIP1^2^ = CB2409; pC2la^1^ = CL313; pC2la^2^ = CB2375, pC2lb^1^ = AVLP567; pC2lb^2^ = AVLP569; pIP14^1^ = AOTU062; pSP7^1^ - SLPpm3_H01; pMP5-pCd2^1^ = SMP286.

We next examined the connectivity between Fru/Dsx subtypes focusing on the strongest connections (using a 3% symmetric threshold; see Methods). 119 out of 23 subtypes (50%) are strongly connected with at least one Fru/Dsx subtype (Fig. 5), and 75 are interconnected in a single connected network, which we term the ‘core’ Fru/Dsx network. First, we observe that all Dsx neurons are in the core network, consistent with the Dsx transcription factor being more conserved across evolution (Kopp, 2012) - we further explore the connectivity of several of the Dsx cells in Fig. 6. In addition, all of the Fru/Dsx DNs (descending neurons) are in the core network, except aSP22. Finally, pC1a and SAG are hubs in the core network (with 8 strong connections for each one of them) - removing them splits the core network into two subnetworks: one that contains most of the Dsx cell types (further explored in Figure 6), the other dominated by pMP3-pCd1, pMP5-pCd2 and aDT6 (see above). Interestingly, the most interconnected subtype in one subnetwork - pMP5-pCd2-SMP286 (with 9 connected edges) gets direct, strong excitation from one of the two most connected types in the other subnetwork (pC1a, 8 edges). This connection was suggested to be involved in several female-specific behaviors (Laturney et al., 2023; Imoto et al., 2024). We also observe that pC1d,e are interconnected with aIPg_b,c, consistent with previous findings on their role in controlling female aggression (Deutsch et al., 2020; Schretter et al., 2020). As recent work in the *Drosophila* visual system suggests that the strongest inputs to a given neuron are the most influential, in terms of physiology (Currier and Clandinin, 2025), the network layout of strongest connections among the Fru/Dsx cell types should prove useful in predicting their function.

### Predicting Function from Connectivity

In an attempt to predict function from connectivity, we focused on the connectivity around two sets of neurons involved in seemingly opposing behaviors: vpoDN neurons, which respond to male pulse song and drive vaginal plate opening, a receptivity behavior (Wang et al., 2021), and DNp13 neurons, which respond to male pulse song (Deutsch et al., 2019) and drive ovipositor extrusion, a rejection behavior (Mezzera et al., 2020; Wang et al., 2020a) (Fig. 6). We built the network in two steps starting from the strongest direct connections of those reception/rejection neurons and any of the Fru/Dsx neurons, then adding a second layer with a higher threshold (see Methods).

By creating a network diagram with strong Fru/Dsx connections around the reception/rejection descending neurons, we identified several interesting properties and motifs, and can predict the function of a new cell type (aSP30). We found that most of the Dsx cells are included in this network, including pC1, pC2l and SAG. Several recent studies had identified functions for these cell types using a mix of neural recordings, activation, or silencing experiments. pC1 neurons promote arousal (Deutsch et al., 2020; Schretter et al., 2020; Wang et al., 2021), pC2l neurons encode male courtship pulse song (Deutsch et al., 2019; Wang et al., 2020a), and the SAG neuron drives female receptivity (Feng et al., 2014). We had previously observed that a large number of neurons in the female brain are tightly tuned to the male’s pulse song (Baker et al., 2022) - here we find that these cell types (pC2la/b, pC1d/e, vpoDN, DNp13, and aSP10) are distributed into different subcircuits for different behaviors (receptivity (vpoDN), rejection (pC2la/b, aSP10a, and DNp13), and aggression (pC1d/e)) (Fig. 6A). This provides a mechanism for how a single syllable of the male’s song can drive so many different behaviors, but it remains to be determined how the brain chooses between these different possibilities as courtship unfolds. The three pC2l subtypes are defined by connectivity (Fig. 4), and we find morphological distinctions between them (Fig. 6B). We also discover that the rejection circuits are multisensory - they integrate pulse song information with visual information via LC31b neurons (Fig. 6C). These visual projection neurons innervate only the posterior portion of the lobula (which receives information from the posterior portion of the visual field, corresponding to the region behind the female), and are prime candidates for processing male motion cues as he chases and sings to her from behind (Fig. 6D); these neurons contrast with LC10a neurons, implicated in female aggression (Schretter et al., 2025), which concentrate their inputs in the anterior lobula (aggression behaviors are driven via frontal motion cues). Females likely combine spatially localized song and visual cues to drive rejection behaviors, such as ovipositor extrusion via DNp13 (Wang et al., 2020a) and wing flicking via pIP9 (Stürner et al., 2025). DNp13 and pIP9 have highly overlapping morphology (Fig. 6E) and connectivity (Fig. 4). Prior work had only found a role for auditory information processing (not visual processing) in rejection behaviors (Wang et al., 2020a). vpoIN neurons are known to respond to pulse songs outside of the conspecific range (Wang et al., 2021); this includes agonistic male song (Hindmarsh Sten et al., 2025). We find that these neurons provide global inhibition to a large number of Fru/Dsx neurons (Fig. 6F), not just vpoDN, as previously studied (Wang et al., 2021; Hindmarsh Sten et al., 2025). This global inhibition may sharpen tuning for conspecific courtship song, in those neurons that are song responsive like pC2la and b, and may act to inhibit all social and sexual behaviors in the presence of male agonistic song or male songs of another species (Tootoonian et al., 2012). Finally, we uncover the function of aSP30 neurons, not previously characterized (Fig. 6G). 29.8% of the input synapses to aSP30 are from the putative pulse song neurons (pC2lb-AVLP567), and 19.7% are direct inputs from the receptivity circuits (pC1a and vpoDN combined). aSP30 inhibits neurons in the rejection and aggression promoting circuits. This cross pathway inhibition motif forms the basis for a behavioral hierarchy (Tinbergen, 1951) - when the receptivity pathway is activated, other behavioral circuits are shut down, giving receptivity a primacy. We do not uncover inhibition in the other direction (from rejection and aggression promoting circuits to the receptivity ones). Taken together, the diagram suggests two major pathways for the control of receptivity and aggression in *Drosophila* females, where multisensory signals, global and cross inhibition shape the chosen behavior.

### Sensory Processing in the Fru/Dsx network

**Figure 7 -:**
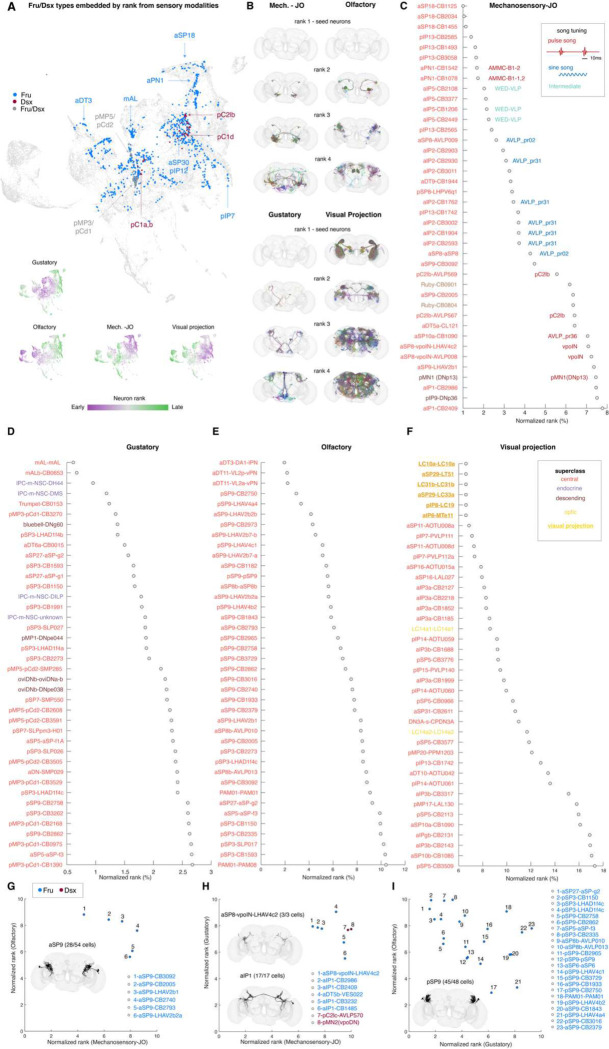
Information flow from sensory modalities to Fru/Dsx neurons (A) Top, UMAP analysis of the matrix of normalized traversal distances (Schlegel et al., 2021; Dorkenwald et al., 2024), resulting in a 2D representation of each neuron in a sensory space (see Methods). Each dot represents a single neuron. Fru, Dsx, Fru-Dsx neurons are colored in blue, red and gray, respectively. The approximated average location of the cells belonging to some types are shown. The Bottom, we colored neurons in the UMAP plot by the rank order in which they are reached from 4 seed groups (Mech. -JO: mechanosensory-JO; see (Dorkenwald et al., 2024)). Purple neurons are reached earlier than green neurons. For example, mAL neurons (see arrow in top panel) are positioned around cells with early rank from the Gustatory sensory neurons (bottom panel ‘Gustatory’), consistent with their role in processing gustatory cues (Clowney et al., 2015), while aDT3 has small rank from the olfactory seed (Jefferis et al., 2007; Datta et al., 2008; Yu et al., 2010), while aSP18 has small rank from the Mech. -JO seed, consistent with its low rank from Mechanosensory-JO (see 7(C); (Liu, 2012)). **(B)** Fru/Dsx neurons with different ranks from different sensory seeds (mechanosensory-JO, olfactory, gustatory, or visual projection; see Methods). No mechanosensory-JO, olfactory and gustatory sensory neurons (rank 1) exist in our Fru/Dsx network, while some visual projection neurons do. **(C)** Fru/Dsx subtypes are sorted by normalized rank from Mechanosensory-JO. Known auditory types (Deutsch et al., 2019; Baker et al., 2022) are colored according to their tuning to syllables of courtship song, ‘pulse’ or ‘sine’. Intermediate turning implies no strong preference to Pulse over Sine song as previously defined (Baker et al., 2022) . **(D-F)** same as (C) for Gustatory (D), Olfactory (E), and Visual projection (F) seeds. Fru/Dsx subtypes are colored by superclass. Normalized ranks from sensory modalities were calculated as before (see Methods), by converting absolute ranks to percentile as previously done (Dorkenwald et al., 2024), and averaged over all the cells in a given subtype. For example, aSP18-CB1125 has a normalized rank of 1% from mechanosensory-JO, implying that 99% of FlyWire cells have a higher absolute rank than an average cell in the aSP18-CB1125 group. **(G–I)** Fru/Dsx subtypes with normalized rank < 10% with pairs of modalities: mechanosensory-JO and olfactory (G), mechanosensory-JO and gustatory (H), gustatory and olfactory (I). Insets show rendered neurons for some multisensory types. Only around half of the aSP9 neurons (28/54) are multisensory (according to their ranks). Compared to all 17/17 (all subtypes) for aIP1 and 45/48 cells (11/12 subtypes) for pSP9. Only one aSP8 subtype (LHAV4c2; 3 cells) is multisensory. Pairs with visual-projection are not shown: no type has normalized rank <10% from both visual proj. and gustatory seeds. Types pSP5-CB3776 and aIP3b-CB1688 have norm. rank<10% with vis. Proj. and norm. rank <10% with olfactory or mech.-JO seeds, respectively.

Prior work used a probabilistic model to estimate information flow in the connectome, from sensory populations through to different intrinsic neurons and out to motor or descending neurons ((Dorkenwald et al., 2024)). This method generated a mapping of every neuron in the central brain (not including the optic lobes) of the connectome relative to the sensory periphery (or sets of sensory seed neurons); Fru/Dsx neurons are distributed throughout this map (Fig. 7A). Here we inspect that ordering to make predictions about the sensory modalities carried by Fru/Dsx neurons (see comments on the interpretation of rank analysis under Discussion). Several of these neurons are proximate to different seed neuron populations (Fig. 7B). By convention, rank 1 cells are defined as cells inside the seed group. While it is known that some sensory neurons are Fruitless-expressing (Manoli et al., 2005; Stockinger et al., 2005; Datta et al., 2008; Dickson, 2008; Yu et al., 2010; Thistle et al., 2012; Toda et al., 2012; Clowney et al., 2015), as stated earlier, we did not include these neurons in our Fru/Dsx list because we could not discern which subsets of sensory neurons are Fru+ based on morphology alone. We calculated a ‘normalized rank’ (Fig. 7C–I) which is a measure for the traversal distance to a given cell from ‘seed’ neurons relative to the distance to all neurons in the connectome from the same seed. When seeding with mechanosensory receptor neurons in the antenna (Johnston’s Organ neurons (JONs) (Kamikouchi et al., 2006; Patella and Wilson, 2018)), we find that aSP18, pIP13, aPN1, and aIP5 are the closest cells types to the mechanosensory periphery (Fig. C). aPN1 and aIP5 neurons have been studied previously (Vaughan et al., 2014; Baker et al., 2022) and shown to respond to male courtship song (in other words, to be auditory neurons of the central brain). For all Fru/Dsx neurons predicted to be mechanosensory (Fig. C), we indicate whether they have previously been shown to be tuned to male pulse song (red), sine song (blue), or to respond to both (teal) (Baker et al., 2022). When seeding from gustatory receptor neurons (Fig. D), mAL neurons are the closest, together with a new type, mALb, that overlaps with mAL morphology but lacks the contralateral projection. mAL neurons have been studied in males for their role in driving arousal following tasting/tapping of the female (Clowney et al., 2015; Kallman et al., 2015) but their role in females is uncharacterized. Other cell types with the lowest gustatory rank include the cell types Prungle, pSG1, Trumpet Foxglove, Horseshoe and Bluebell, all previously characterized as taste neurons (Tran et al., 2014; Sterne et al., 2021). The proximity of oviDN neurons (Wang et al., 2020b) to the taste periphery suggests a mechanism by which taste information influences egg laying decisions. When seeding from olfactory receptor neurons, Fru aDT3 and aDT11 uniglumerular PNs (projection neurons) in the antennal lobe are the closest in rank. The lowest olfactory rank neurons are aDT3 (well-known, DA1 lateral projection neurons), Fru+ projection neurons (PNs) that project to the anterior ventral region of the lateral horn and respond to the male-specific pheromone cVA (Taisz et al., 2023). Like aDT3, aDT11 (also called VL2a_vPN) innervates a sexually dimorphic glomerulus (Stockinger et al., 2005). While VL2a ORNs sense food odors, VL2a PNs project axons to the pheromone-specific regions of the lateral horn, and function in promoting mating behavior (Jefferis et al., 2007; Grosjean et al., 2011). Other types with low rank distance from the olfactory seed are aSP8b, aSP9, pSP3, pSP9 (sharing morphology with pSP-g, aIP-e and pSP-d and pSP-f (Cachero et al., 2010), respectively). Those cell types all innervate the lateral horn (LH), an area proposed to mediate innate olfactory behavior, odor categorization (Frechter et al., 2019) and multisensory integration (Dolan et al., 2019). Those Fru types are heterogeneous groups, with varying connectivity with the rest of the Fru/Dsx network. pSP9-LHAV4a4, for example, has 58%/49% of its input/output synapses with Fru/Dsx cells (excluding within subtype connectivity; see Fig. 3) and has a normalized rank of <4% from the olfactory seed.

To characterize visual neurons, we started from the visual projection neurons - visual neurons that carry information from the optic lobes to the central brain (Wu et al., 2016). Several Fru neurons are visual projection neurons themselves (LC10a, aSP29-LT51, LC31b, aSP29-LC33a, pIP8-LC19, and aIP6-MTe11). And we identified two Fru optic lobe neurons (LC14a1 and LC14a2) that are close to the VPNs. Other cell types found to be close in rank to the VPNs are aSP11 (AOTU008a,b) - the AOTU contains the processes of LC10a, known to be involved in male courtship and female aggression (Ribeiro et al., 2018; Hindmarsh Sten et al., 2021; Schretter et al., 2025), pIP7, which is directly postsynaptic to LPLC2 (which detects looming stimuli). aIP3a is postsynaptic to LC16. Many LC neurons are involved in male courtship of the female (Cowley et al., 2024), and it will be interesting to examine how Fru LCs and non-Fru LCs integrate social information to drive behavior via these cell types. Overall, we find that Fru/Dsx cells are closer to the mechanosesory, gustatory and olfactory seeds, and further from visual projection (VPN) seeds. It is important to note that rank distance is a probabilistic measure, and cells with high rank from a given modality may still be weakly connected directly to a cell in the ‘seed’ neurons of this modality. For example, aSP9-LHAV2b2a receives weak direct input from LC43, but does not have low normalized rank from the visual projection neurons (Fig. 7F).

Social behaviors in *Drosophila* often involve integration of sensory cues of different modalities (Dickson, 2008; Zhou et al., 2014; Clowney et al., 2015; Rings and Goodwin, 2019). Here, we examine proximity to more than one ‘seed’ modality. We find Fru/Dsx neurons that are close to both olfactory and mechanosensory receptor neurons (Fig. 7G), to both gustatory and mechanosensory receptor neurons (Fig. 7H), and to both olfactory and gustatory receptor neurons (Fig. 7I). There were no visual-olfactory neurons (with ranks <10%, see Methods), only 1 visual-gustatory cell type (pSP5-CB3776) and 1 visual-mechanosensory cell type (aIP3b-CB1688). aSP9 neurons are predicted to process both olfactory and mechanosensory information: they primarily receive inputs in the lateral horn (LH; containing third order olfactory neurons) and project to the AVLP and PVLP; all three brain areas are known to carry auditory signals (Pacheco et al., 2021). aSP8 and aIP1 neurons are both predicted to process gustatory and mechanosensory information. aSP8 has previously been shown to carry auditory (pulse song) responses (Wang et al., 2021; Baker et al., 2022), but a role in taste processing has not been investigated. aSP8 is activated by male agonistic songs (Hindmarsh Sten et al., 2025), and it may combine this information with contact pheromones from males to inhibit receptivity in females (Wang et al., 2021). aIP1 neurons have not been investigated previously. pSP9 neurons in the lateral horn are predicted to process both olfactory and gustatory information, but only olfactory coding has been studied in these neurons (Jefferis et al., 2007; Kohl et al., 2015; Jeanne et al., 2018). Taken together, this analysis points to strong multisensory coding among several Fru/Dsx neurons.

### Connectivity With Descending Neurons

**Figure 8 -:**
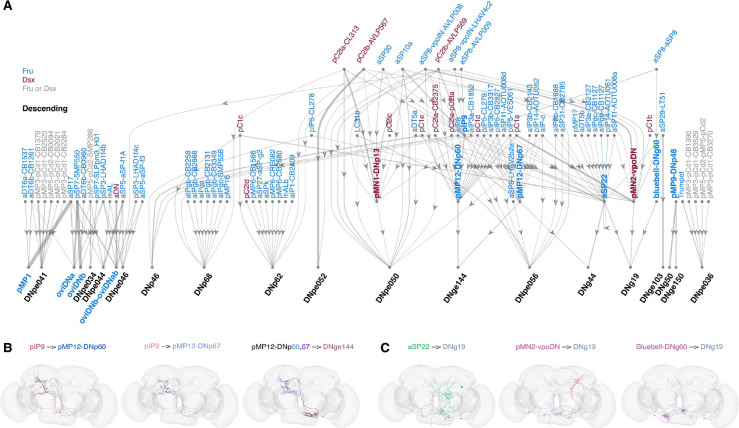
Fru/Dsx neuron connectivity to descending neurons **(A)** Strong direct synaptic connections (1% or larger, symmetric threshold; see Methods) between Fru/Dsx neurons and DNs (including DNs that are Fru/Dsx, such as pMN1-DNp13 and pMN2-vpoDN and DNs that are not in the Fru/Dsx list). This map reveals DNs that may be important for driving sexually dimorphic behaviors. Some of these have already been characterized (for example, Fru oviDNs or Dsx vpoDN (Wang et al., 2020b, 2021; Vijayan et al., 2023)), but most have not. Fru, Dsx, Fru-Dsx types are colored blue, brown and gray as elsewhere. Descending neurons not in the Fru/Dsx list are marked in black. Possibly some of these types are Fruitless or Doublesex positive. All Descending neurons are marked with an underline. Note that some descending neurons are upstream of other descending neurons, and are therefore not at the bottom row of the map. **(B)** Examples for connections between pairs of descending cells: fru+pIP9 is upstream of both pMN12-DNp60 (left), and pMP12-DNp67 (middle). Both pMP12-DNp60 and pMP12-DNp67 are directly upstream of DNge144, a pair of descending neurons (one per hemisphere) that are not in the Fru/Dsx list. Synapses are shown as red circles. (C) Three Descending types have strong direct connections upstream the descending cell type DNg19 (which is not in the Fru/Dsx list): fru+aSP22 (left), dsx+pMN2-vpoDN (middle) and fru+Bluebell-DNg60. Synapses are marked in yellow.

In *Drosophila* and other insects, the neck connective is both a physical and information bottleneck connecting the brain and the ventral nerve cord (VNC; spinal cord analogue) (Namiki et al., 2018; Chen et al., 2023; Braun et al., 2024; Stürner et al., 2025). A number of descending neurons (DNs; which carry information from the brain to the VNC) and ascending neurons (ANs; carrying information from the VNC to the brain) have been identified as Fru/Dsx previously and associated with sexual or social behaviors. For example, the sexually-dimorphic Dsx pMN1 (DNp13) and the female-specific Dsx pMN2 (vpoDN) neurons control female ovipositor extension (Mezzera et al., 2020; Wang et al., 2020a) (female DNp13) and female vaginal plate opening (Wang et al., 2021) (female vpoDN). Activation of the sexually-dimorphic Fru aSP22 DN triggers a sequence of close-range courtship actions in males, including front leg extension, proboscis extension and abdominal bending (McKellar et al., 2019). Leg movements and abdomen movement are also elicited in females (McKellar et al., 2019), but while males bend their abdomen in response to aSP22 activation, females extend it, possibly a behavior related to another behavior. The female-specific Dsx SAG ascending neurons respond to male-transferred sex peptide and relay information to brain Dsx pC1a neurons controlling female receptivity (Feng et al., 2014; Wang et al., 2021). We identified in total 2 ascending (1 type) and 28 descending (11 types) Fru/Dsx cells. The descending neurons comprise 14 connectome-based subtypes (e.g., DNs aSP3, oviDNb and pMP12 have 2 subtypes each). We next mapped direct connectivity between all Fru/Dsx subtypes (Fig. 1–2) and any descending neuron in the whole-brain connectome, focusing on the strongest connections (Fig. 8A; see Methods). We find that 29 DN types have 10% or more of their input synapses with Fru/Dsx neurons (including Fru/Dsx DNs). Only one Fru/Dsx type is missing from the diagram: aSP3 (DNa13_a,b); this is because it does not match the threshold of 10% inputs from Fru/Dsx neurons to a DN applied for this figure.

Examining the network of DNs connected to Fru/Dsx neurons reveals several interesting features. First, while we do not yet know the function of DNpe034, 041, 044, and 046, their clustering with DNs involved in oviposition (pMP1, oviDNa, and oviDNb) implicates them in this process. pC1d and aIPg neurons have been previously studied for their role in female aggression (Deutsch et al., 2020; Schretter et al., 2020; Chiu et al., 2023); here, we see that these neurons converge onto DNp68, implicating it in aggressive behaviors. DNp68 also receives input from pC2la-CL313, predicted to be responsive to the male’s courtship pulse song (Figs. 6–7;(Deutsch et al., 2019)), indicating that perception of male song can drive both receptivity (via vpoDN) and aggressive behaviors. There is some precedent for this, as pC1d and pC1e neurons that together drive a persistent aggressive state in females (Deutsch et al., 2020; Chiu et al., 2023), and are both tuned to the male’s pulse song (Deutsch et al., 2019). DNp13 is a descending neuron that also responds to male pulse song and drives ovipositor extension, a rejection behavior ((Deutsch et al., 2019; Wang et al., 2020a).; Fig. 6); it also receives input from pC2la and b neurons. Through this analysis, we find it receives similar inputs as DNpe050 and 052, DNs not yet characterized. We find that pC1a, which drives vpoDN, a DN required for receptivity to male song (Wang et al., 2021), also drives pMP12-DNp60, implicating this new cell type in receptivity. aSP22 receives strong direct excitatory inputs from aIPgb,c - cell types involved in controlling female aggression (Deutsch et al., 2020; Schretter et al., 2020)), raising the possibility that aSP22 is involved in controlling female aggressive behaviors. We identified asp30 in this study as a cross-pathway inhibitory neuron (Fig. 6) - it receives excitatory input from neurons involved in receptivity and inhibits neurons involved in rejection (like DNp13). Here, we find that asp30 also inhibits both DNpe050 and 052, further implicating both in rejection behaviors. One final property observed here, though not specific to Fru/Dsx connectivity, is that several DNs have other DNs as one of their major inputs (Fig. 8B–C). This type of DN-to-DN connectivity has been recently analyzed functionally (Braun et al., 2024) and is likely important for recruitment of motor command pools to drive behaviors. Once a full CNS (brain and nerve cord) connectome is completed, it will be possible to interpret the connections shown in Figure 8, relative to the outputs of these DNs onto the motor circuits of the VNC.

A total of 17 sexually dimorphic DN types with identified projections in FAFB/FlyWire were recently reported based on the comparison of male and female nerve cord connectomes (Stürner et al., 2025). This included 10 sexually-dimorphic types (5 of these are in our list: Dsx DNp13 (Wang et al., 2020a) and Fru aSP22 (McKellar et al., 2019), pIP9 (Yu et al., 2010; Lillvis et al., 2024), oviDNx/pMP1 (Wang et al., 2020b), and DNp48) and 7 female specific types of which 4 are included in our list (Dsx vpoDN (Wang et al., 2021), Fru oviDNa,b,a_b (Wang et al., 2020b)). Sex-specific cell types not included in our list include DNpe044, DNpe046 and DNpe047 (also called oviDNd,v,x). Interestingly, of those three types, two (DNp044, DNpe046) were identified as receiving a large fraction of synapses from Fru/Dsx neurons (Fig. 8A). Three Fru/Dsx types in our study were not reported as sexually dimorphic in (Stürner et al., 2025): the SEZ Bluebell (DNg60) neurons (Sterne et al., 2021) and two new types (aSP3 (DNa13) and pMP12 (DNp60, DNp67) (see Fig. 2S1)). aSP3 has overlapping morphology with DNa08 (Stürner et al., 2025), but comparing the VNC projection of aSP3 in (Yu et al., 2010) to (Stürner et al., 2025) reveals that this cell type matches DNa13 better. Taken together, this comparison reveals the complementary power of using our approach (LM-EM comparison in one sex) and EM-EM comparison of male-female connectomes in identifying sexually dimorphic circuits.

## DISCUSSION

In this study, we provide the first comprehensive mapping of putative *Fruitless* (Fru) and *Doublesex* (Dsx) neurons in the female Drosophila whole-brain connectome, identifying 91 Fru/Dsx types comprising over 1400 neurons. By combining light microscopy (LM) datasets with the FlyWire electron microscopy (EM) reconstructions, we establish a morphologically defined dataset of putative sexually dimorphic neurons and examine their connectivity patterns across the brain. This work offers insight into how Fru/Dsx circuits are embedded within broader neural networks and enables predictions about their function based on connectivity.

### Limitations of the Current Analysis

While extensive, our analysis captures only a subset of Fru/Dsx neurons in the female brain. Several neuron classes remain underrepresented or excluded, such as Kenyon cells of the mushroom body (*fru P1* is expressed in the mushroom body γ and αβ KCs (Billeter and Goodwin, 2004; Pan and Baker, 2014; Palmateer et al., 2023)), optic lobe neurons with ambiguous Fru expression (Chiang et al., 2011; Tanaka et al., 2017), and sensory neurons whose morphology does not allow reliable Fru/Dsx classification. The neurons included in our dataset are chosen solely on the basis of their morphological overlap with LM images, and it is likely that some neurons in our list do not express the *Fruitless* or *Doublesex* genes. We divided the 91 Fru/Dsx types into subtypes, based on morphology and connectivity, and the fraction of input/output synapses each subtype has with any other Fru/Dsx neuron (Fig. 3). Based on the observation that Fru/Dsx neurons tend to connect to each other, the fraction of connections with the Fru/Dsx network can serve as an indicator for which subtypes, within a given type, are more likely to be Fru/Dsx expressing, or at least - to be part of the circuits involved in controlling social behaviors in flies. Furthermore, due to the limited resolution in distinguishing Fru from Dsx expression based on morphology alone, some cell types, particularly pMP3/pCd1 and pMP5/pCd2, are grouped together. While based on previous literature and the FlyWire connectome we can likely distinguish between pMP5 and pCd2, future work involving antibody staining for Fru and Dsx proteins followed by EM reconstruction could yield a more definitive identification of these neurons and help disambiguate overlapping types.

### Toward a Comparative Connectomics of Sexual Dimorphism

A major next step will be to compare this female connectome to an equivalent male whole-brain connectome (as was previously done in worms (Cook et al., 2019)). Such comparisons would allow identification of male-specific neurons (e.g., P1, aSP2, and others), female-specific neurons, and neurons with sex-differentiated cell number, morphology and connectivity. However, even with the forthcoming availability of a male whole-brain connectome, distinguishing sex differences from inter-individual variability remains a challenge. Moreover, some Fru/Dsx neurons, despite expressing sex determination genes, may not have only subtle differences in morphology and connectivity. In this context, our approach of mapping Fru/Dsx neurons via morphological matching provides a complementary strategy to connectome comparison, offering hypotheses grounded in well-validated LM datasets.

### Implications for Understanding Female Behavior

Sexually dimorphic behaviors in *Drosophila*, including courtship, receptivity, aggression, and egg-laying, rely on distinct but overlapping neural circuits (Hoopfer et al., 2015; Koganezawa et al., 2016; Deutsch et al., 2020; Hindmarsh Sten et al., 2025). Prior work has focused more on male-specific behaviors, such as courtship chasing (Kohatsu and Yamamoto, 2015; Ribeiro et al., 2018; Hindmarsh Sten et al., 2021; Mabuchi et al., 2023) and singing (von Philipsborn et al., 2011; Shirangi et al., 2013; Roemschied et al., 2023; Lillvis et al., 2024). By anchoring Fru/Dsx neurons within a complete female connectome, our work illuminates the wiring logic of female social behaviors and opens new avenues for targeted functional experiments. Ultimately, this work lays the foundation for a functional connectome of sexual and social behaviors in Drosophila. It enables researchers to move beyond isolated circuit fragments and toward a holistic understanding of how sensory inputs are integrated by Fru/Dsx neurons, transformed through local processing, and relayed to descending motor pathways. As larger datasets, including multiple brains and sex-specific connectomes, become available, the hypotheses and network motifs described here will serve as a critical scaffold for understanding how evolution sculpts the nervous system to generate sex-specific behaviors.

## METHODS

### Identifying Fru/Dsx cells in FlyWire

Matches for Fru/Dsx cells were found in FlyWire (version 783) based on morphological similarity to light microscopy images. The light microscopy sources included both light microscopy stacks of sparse driver lines or stochastic labeling of fru-Gal4 or Dsx-LexA lines (see Table 1). In order to find putative fru candidate cells in FlyWire, we used image-based matching from the FlyCircuit (Chiang et al., 2011) and Cachero et al. (Cachero et al., 2010) clones available as light microscopy image collections. We obtained the collections of light microscopy images by downloading them from Virtual Fly Brain (https://www.virtualflybrain.org/). We performed alignment of the FlyCircuit images from the FCWB to the JRC2018U template space using navis-flybrains (https://github.com/navis-org/navis-flybrains/). The Cachero et al. images were already available in the JRC2018U template space. We then generated Color Maximum Intensity Projections (ColorMIP) images from binarized version of those clone images (Otsuna et al., 2018) in a new Python implementation (https://github.com/htem/FANC_auto_recon/blob/main/fanc/render_neurons.py). Then, we ran image-based matchings against the collection of ColorMIP images for FlyWire v783 (as in (Otsuna et al., 2018)) to infer a ranked list of FlyWire candidate neurons for each clone. We then manually reviewed the candidate matchings with automatically generated Neuroglancer links showing the FlyWire candidates together with the corresponding mesh extracted from the binarized version of the clone image to arrive at the final set of matches for further analyses. Some of the electron-microscopy matches were first identified in the hemibrain (Scheffer et al., 2020), and then mapped to FlyWire using FlyWire Gateway (https://flywiregateway.pniapps.org/hemibrain_neurons), which was also used for finding candidates in the contralateral hemisphere. The Gateway candidates were manually sorted by comparison to the light microscopy images. Once FlyWire matches were identified for all candidates, we used NBLAST (Costa et al., 2016) to look for more candidates. To further extend our search we next used similarity in connectivity to find more candidates. Last, left-right symmetry was used both for perfecting the proofreading (by identifying missing or extra branches on one hemisphere compared to the other one). The division into subtypes was done based on existing clustering into cell types that are largely defined by connectivity ((Matsliah et al., 2024; Schlegel et al., 2024)); see Table 1). It is likely that some subtypes are Fru/Dsx negative (see Figure 3).

### Matched networks

In order to compare the connectivity of Fru/Dsx cells against populations of cells with similar properties we created 100 samples of ‘matched networks’. Matched networks were assembled as follows: (1) The same number of cells per superclass as in the Fru/Dsx group (Fig. 1G) was randomly chosen from all the FlyWire cells, excluding the Kenyon cells (as Fru/Dsx kenyon cells were also excluded). (2) The pairwise euclidean distances between all the possible pairs of randomly selected cells was computed. If the mean distance was similar to the mean pairwise distance within the Fru/Dsx network (±2%), this group was selected as one a ‘matched network’; if not it was rejected. Distances are measures between cell bodies. (3) This procedure was repeated until 100 matched networks were selected.

Note that while the mean pairwise distance in the Fru/Dsx network is 210μm and the mean distance over groups of FlyWire cells (no kenyon cells, same superclass distribution) is 208.1 (<1% difference), most of the random networks are not within 2% of the Fru/Dsx mean pairwise distance due to the variability in the mean distances across random networks (±11.1μm).

### Measuring the bias for number of synapses with Fru/Dsx partners

Connections between proofread neurons are thresholded with 5 synapses for all the analyses described in this paper as previously done (e.g., (Dorkenwald et al., 2024; Lin et al., 2024)). We aimed to reveal if Fru/Dsx cells are biased to have more synapses with a Fru/Dsx synaptic partner compared to the number of synapses they have with a random synaptic partner (Fig. 1S3). We measured this bias separately for Fru/Dsx inputs and outputs. The procedure described here is for the inputs, a similar procedure was used for the outputs.

For each Fru/Dsx cell we made two calculations. First, we calculated the total number of input synapses it has with all its Fru/Dsx input partners (N_DsxFru, a single value)). Second, we made 100 random choices of N input partners out of all the input partners of this cell (N being the number of Fru/Dsx input partners), and calculated the number of input synapses for this group (V_Rand, a vector with 100 values). Then we calculated the normalized bias (Fig. 1S3) as (N_DsxFru-mean(V_Rand))/std(V_Rand), where ‘std’ stands for standard deviation. Positive/negative values mean a bias towards more/less synapses with Fru/Dsx partners, respectively.

### Symmetric threshold’ for direct synaptic connectivity

For network analysis, In order to visualize only the strongest connections, we applied a ‘bidirectional threshold’ with different thresholds being used for different visualizations (Figs. 5,6). For example, with a bidirectional threshold of 3%, a connection from group type A and group type B is shown if and only if two criteria are met. First, at least 3% of the input synapses from all the cells in group B are from cells in group A and at least 3% of the outputs from all the cells in group A are with cells in group B.

### Clustering of the Fru/Dsx subtypes

A 3% symmetric threshold was applied, leaving only the strongest connections between Fru/Dsx pairs. 119 subtypes out of 236 are present in the network. Then, for the 119 subtypes, we used hierarchical clustering to group Fru/Dsx subtypes into connectivity clusters (Fig. 4). First, we calculated for each Fru/Dsx subtype *t,* a feature vector of length 2T, where T is the number of cell types in the FlyWire connectome. The first T values in the vector represent the number of synapses that cells of type t receive from every upstream type. The last T values in the feature vector count the number of synapses sent to each downstream type. Then we calculated distances between all pairs of Fru/Dsx subtypes as L2 norm between their corresponding feature vectors. Then we applied hierarchical clustering with average linkage on the calculated distance matrix, and cut at distance 0.3 to define the connectivity clusters.

### Connectivity diagram of the Fru/Dsx types

In the connectivity diagram for Fru/Dsx types (Fig. 5), we show an edge from type A to type B only if at least 3% of A’s output synapses are to B cells **and** at least 3% of B’s input synapses are from A cells. Roughly speaking, we show edges between pairs of types only if they are mutually important to each other. Edge widths further represent the absolute count of synapses connecting the types, and edge tips represent action (round=inhibition. arrow=excitation). Node sizes are proportional to the number of connections, their colors represent superclasses, and their shapes represent genes (ellipse=Dsx, octagon=Fru, rectangle=Dsx or Fru, hexagon=other). The visualization was made with Cytoscape, and the layout was initialized using yFiles organic layout with manual adjustments.

### Rank analysis - information from from sensory modalities

The traverse distance is a probabilistic measure for the number of hops from a group of neurons (the ‘seed’ neurons) to any individual neuron as defined in (Schlegel et al., 2021). The distance from the seed group to any other cell is calculated multiple times and then averaged. The average value is defined as the cells ‘rank’ with respect to the seed group. The distance is calculated probabilistically in the following way (as in (Schlegel et al., 2021)). We start with an initial pool of cells called the ‘seed’ neurons. Then on each step, cells are added to the pool, where the probability P of a not-yet-traversed neuron to be added to the pool depends on the fraction of the inputs it receives from neurons already in the pool. For ‘fraction inputs’ between 0 and 0.3, P is the fraction divided by 0.3. For ‘fraction inputs’ of 0.3 or more, P=1. The number of steps it takes for a cell to be added to the pool is its traverse distance. This procedure is repeated multiple times (10000 times when we calculated the distance from sensory modalities and 1000 times when Fru/Dsx cell types were used as seeds) and then averaged the distance over repetitions. The ‘rank’ is calculated as 1+mean traverse distance, such that the cells in the seed group get a rank of 1 and the rest of the cells get a rank > 1. The cells included in each rank distance from 3 groups of sensory neurons (Mechanosensory-JO, gustatory, olfactory; (Dorkenwald et al., 2024)) and from visual projection neurons (Dorkenwald et al., 2024) are shown in Fig. 7B . We normalized the rank to percentile to get a ‘normalized rank’ as in (Dorkenwald et al., 2024): the relative distance of a cell from the seed. For example, if a cell has a normalized rank of 20%, it means that 80% of the FlyWire cells have a larger rank from this Fru/Dsx type/seed (Fig. 7C–I). Small normalized rank means - shorter rank distance from the ‘seed’.

### Direct connectivity to descending neurons

First, connections between proofread neurons are thresholded with 5 synapses as for all the analyses described in this paper. Second, we identified all the descending types in FlyWire (Stürner et al., 2025) that have at least 10% of their input synapses with Fru/Dsx neurons. For those descending neurons, any connection between a Fru/Dsx subtype and a descending type is shown.

## Supplementary Material

Supplement 1

Supplementary Files

This is a list of supplementary files associated with this preprint. Click to download.

Table1FruDsxcells.xlsx

Table2FlyWirelinks.xlsx
