# Supplementary material for: Sexually-dimorphic neurons in the Drosophila whole-brain connectome
Source: Res Sq. 2025 Jun 26:rs.3.rs-6881911. Preprint. [Version 1] doi: 10.21203/rs.3.rs-6881911/v1 (PMC12270219; doi:10.21203/rs.3.rs-6881911/v1)
Supplement: Supplement 1 [file NIHPPrs6881911v1-supplement-1.pdf]

Supplementary Figures

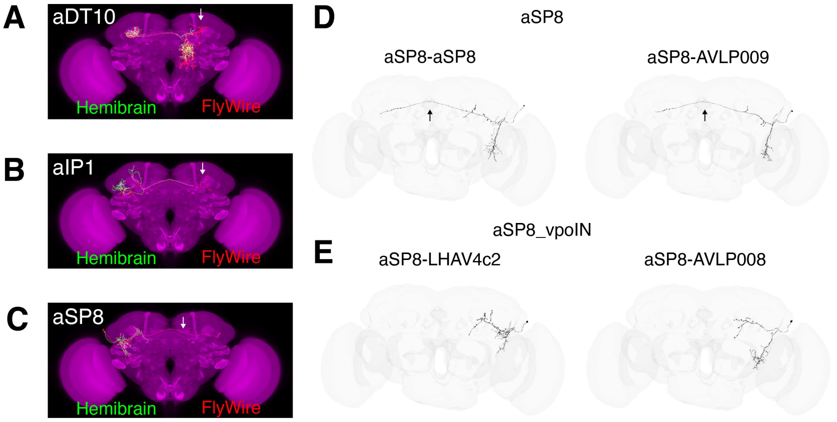

Supp Figure 1 - S1 Comparison of Fru/Dsx types in hemibrain and FlyWire wiring diagrams **(A)** Fru/Dsx aDT10 neurons in the FlyWire ((Dorkenwald et al., 2023); red) and hemibrain ((Scheffer et al., 2020); green) dataset. The two morphologies overlap, but the left lateral projection (ipsilateral to the soma; white arrow) is outside the hemibrain coordinates, and therefore missing in the aDT10 neurons in the hemibrain. **(B)** aIP1 in FlyWire and hemibrain datasets. The contralateral projection (white arrow) is missing in the hemibrain. **(C)** aSP8 in FlyWire and hemibrain datasets. The contralateral projection (white arrow) is missing in the hemibrain. **(D)** aSP8 and aSP8_vpoIN have overlapping morphologies, but aSP8_vpoIN neurons (previously annotated as vpoIN (Wang et al., 2021)) are missing the contralateral projection (see black arrows). Based on this morphological difference, aSP8 and aSP_vpoIN are considered two distinct Fru/Dsx types. 'aSP8' and 'aSP8_vpoIN' have 2 subtypes each. Subtypes are based on a combination of morphology and connectivity (Schlegel et al., 2024) and are shown as cell types in CODEX (Matsliah et al., 2023).

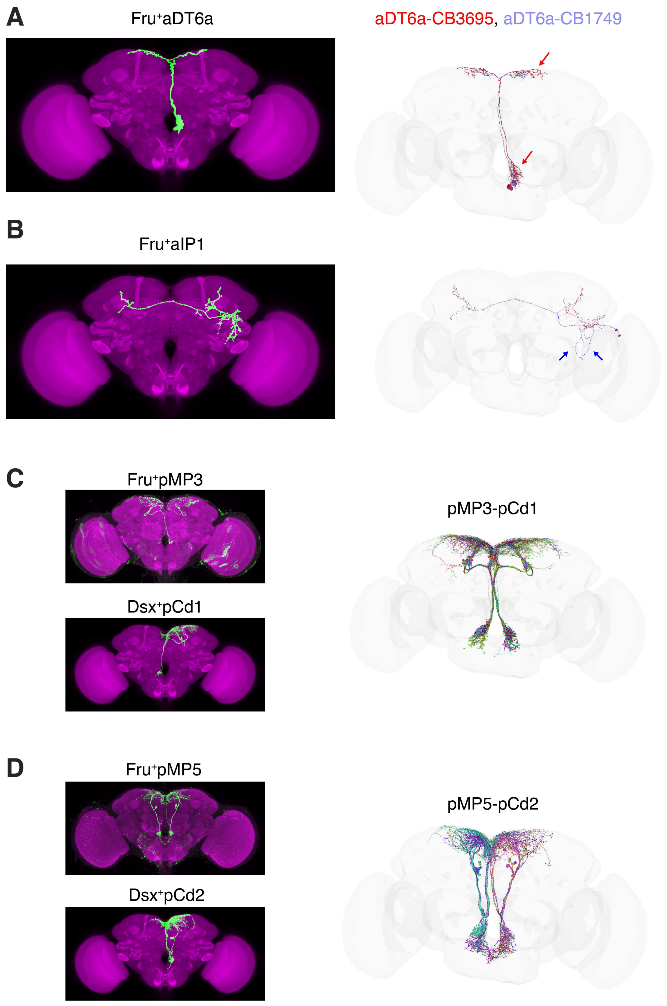

Supp Figure 1 - S2 (A,B) Fru/Dsx types, defined by their morphology in light morphology images, can be composed of multiple subtypes in FlyWire. Subtypes may differ in subtle morphological details. **(A)** aDT6a, LM (left) and EM (right) images. Red arrows indicate dorsal-lateral projections and denser projections near the soma for aDT6a-CB3695 (red) compared to aDT6a-CB1749. **(B)** ventral branches are longer in aIP1-CB2409 (blue arrows) compared to CB1485. Both subtypes match the LM image (left) well,**(C,D)** Fru+pMP3 has similar morphology as Dsx+pCd1, and Fru+pMN5 has similar morphology as Dsx+pCd2. Therefore, in this case, one group of FlyWire neurons potentially contains two Fru/Dsx cell types. **(C)** Similar morphology for pCd1 and pMN3. This type in FlyWire is termed pMP3-pCd1. **(D)** same as in (C), for pMP5-pCd2.

Supp Figure 2 - S1 - Summary cards for Fru/Dsx subtypes
Fru/Dsx summary cards:
https://drive.google.com/drive/folders/1EEjvmwRRhtN1uH44AzizTniGnzRfsvUL?usp=sharing

For each Fru/Dsx subtype a single card is created. For example, for the Dsx expressing pC2lb type, two subtypes/cards are included: pC2lb-AVLP567, pC2lb-AVLP569. Each card includes the following information: Number of cells (total and per hemisphere), Predicted neurotransmitter. render of all the cells included in the subtype, summary of top input and output types, main input/output neuropil (Ito et al., 2014), average number of synapses per cell for inputs and outputs and percentile rank for 7 modalities (see Methods). For input/output partners, filled/empty kite indicates a type included/not-included in our Fru/Dsx list, respectively. Neurotransmitter type is based solely on (Eckstein et al., 2024) for consistency with https://codex.flywire.ai even when the predicted neurotransmitter is different from a previously reported neurotransmitter. For example, the SAG neurons were revealed to be cholinergic by Fluorescent in situ hybridization (FISH) (Wang et al., 2020b), while predicted as Serotonergic in EM-based classification (Eckstein et al., 2024).

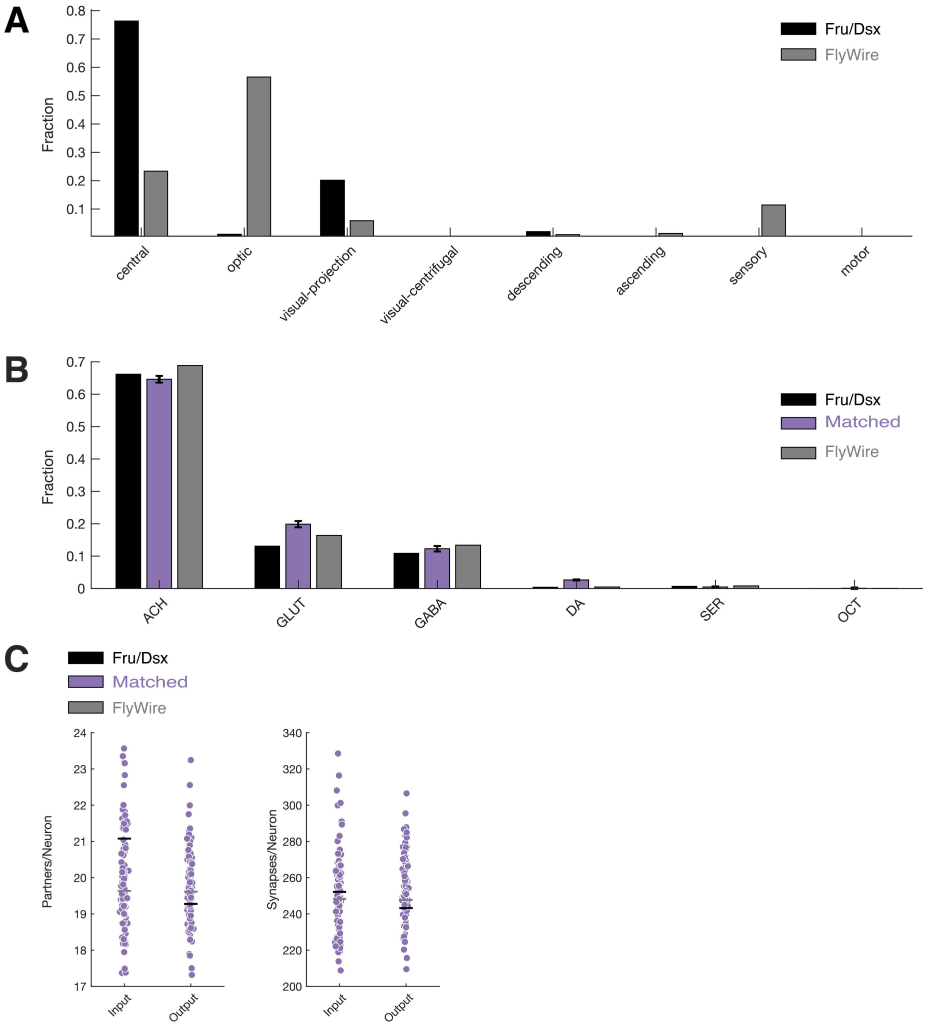

Supp Figure 3 - S1 Comparing Fru/Dsx and 'Matched' Networks
(A) Distribution of Fru/Dsx neurons (black) and all FlyWire neurons (gray) across 'superclasses' (Dorkenwald et al., 2024). **(B)** Distribution of predicted neurotransmitters for all FlyWire neurons (gray), for Fru/Dsx neurons (black), and for neurons in the 100 matched networks (purple). **(C)** Mean number of synaptic partners per neuron (left) and number of synapses per neuron (right) for Fru/Dsx neurons (black), all FlyWire neurons (gray) and for each of 100 matched networks (purple).

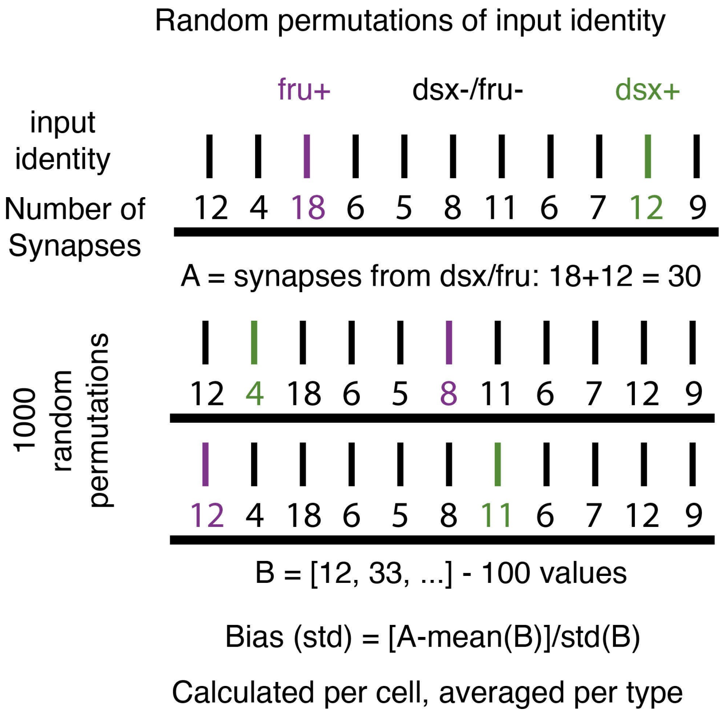

Supp Figure 3 - S2
Calculating the bias for having more or less synapses with Fru/Dsx partners
Illustration of the calculation done in Fig. 3C (see Methods for details). The tendency of Fru/Dsx neurons to have more or less synapses with Fru/Dsx partners compared to the number of synapses with random partners is calculated separately for input and output synapses (here illustrated only for the inputs).
In short, for each cell in the Fru/Dsx list, the total number of synapses with Fru/Dsx neurons is calculated (30 in this example). Then, the identity of the partner neurons as being Fru+ (blue), Dsx+ (red) or not Fru/Dsx (black) is shuffled 100 times and each time the total number of synapses is calculated for the 'Fru'/'Dsx' partners. Then, the bias is calculated as the difference between the real number of the synapses with Fru/Dsx partners (A), minus the mean number of synapses with 'Fru'/'Dsx' partners (B), divided by the standard deviation of B. The average bias is calculated over all the cell in any Fru/Dsx type. The distribution of the biases over the cell types is shown in Fig. 3C.

Tables

Table 1 - Fru/Dsx cells

Table 2 - FlyWire links
